# Resistance to morphine analgesia and its underlying mechanisms in an experimental mouse model of fibromyalgia

**DOI:** 10.1186/ar3672

**Published:** 2012-02-09

**Authors:** Hitoshi Uchida, Michiko Nishiyori, Hiroshi Ueda

**Affiliations:** 1 Division of Molecular Pharmacology and Neuroscience, Nagasaki University Graduate School of Biomedical Sciences, Nagasaki 852-8521, Japan

## 

Fibromyalgia (FM) is a common condition with generalized or widespread allodynia that affects at least 2% of the US, European and Japanese populations. Although the etiology of this disease remains poorly understood, physical and psychological stressors have been assumed to play a role in the development of FM. Previously, we have established an experimental mouse model of FM pain, using intermittent cold stress (ICS) exposure. This model was found to produce mechanical allodynia and thermal hyperalgesia in a female-predominant manner, as often observed in FM patients. In contrast, exposure to constant cold stress produced a transient allodynia. Importantly, we found that anticonvulsant agent gabapentin, especially when injected intracerebroventricularly, exerts powerful anti-allodynic and anti-hyperalgesic effects in the ICS-exposed mice. In this study, we found that ICS model mice show morphine resistance, as often observed in FM patients. To be concrete, systemic or intracerebroventricular, but not intrathecal or intraplantar, injection of morphine caused no significant analgesia in the ICS-exposed mice. In addition, we found that intracerebroventricularly administrated morphine increases the 5-hydroxytryptamine turnover ratio in the dorsal half of the spinal cord of control mice, but not in the ICS-exposed mice. These findings indicate that ICS model well reflects pathological and pharmacotherapeutic features of FM pain, and the loss of descending serotonergic activation seems to be a crucial mechanism underlying the absence of morphine-induced analgesia in the ICS model.
